# Dose-Painting Linear Accelerator Radiosurgery of Glomus Jugulare With Dosimetric Comparison to Gamma Knife

**DOI:** 10.7759/cureus.55070

**Published:** 2024-02-27

**Authors:** Alessandro Valderrama, Long Di, Elizabeth Bossart, Adrien A Eshraghi, Eric A Mellon

**Affiliations:** 1 Radiation Oncology, University of Miami Sylvester Comprehensive Cancer Center, Miami, USA; 2 Neurological Surgery, University of Miami Miller School of Medicine, Miami, USA; 3 Otolaryngology, University of Miami Miller School of Medicine, Miami, USA

**Keywords:** glomus jugulare, paraganglioma, jugular fossa, radiotherapy, linear accelerator, facial nerve, dose-paint, hyperarc, gamma knife

## Abstract

Objectives

In this study, we outline our rationale for delivering a dose of ≥15 Gy in stereotactic radiosurgery (SRS) of glomus jugulare tumor (GJT) while ensuring the avoidance of complications associated with doses >13 Gy to the facial nerve. To avoid such complications, we initially utilized the Gamma Knife Perfexion (GK) system (Elekta Instrument AB, Stockholm, Sweden) at our institution but encountered challenges related to lengthy treatment times and difficulty in sculpting doses to minimize doses to spare the facial nerve. As a potential solution, we propose the use of HyperArc (Varian Medical Systems, Palo Alto, CA), a newly developed automated delivery platform for linear accelerator (LINAC)-based SRS. HyperArc offers the potential for faster treatment and more complex shaping of the radiotherapy dose with multiple arcs and multi-leaf collimators.

Methods

We retrospectively reviewed nine cases of patients with GJT treated with HyperArc. Patients’ demographic and treatment data were collected. Additionally, simulated GK treatment plans were created and compared with HyperArc plans to assess time savings, PTV coverage, and plan quality.

Results

One male and eight female patients, with a mean age of 63.9 years, were included. Treatments were delivered on average in 29 minutes, achieving 95-100% of the tumor while limiting the facial nerve to <13 Gy. Treatments replanned using our GK system could achieve only 92-99% tumor coverage while respecting facial nerve constraints, with average treatment times of 180 minutes. Comparable plan quality parameters were attained with both modalities.

Conclusions

The HyperArc system provides a qualitatively satisfactory and rapid treatment delivery of a highly sculpted radiotherapy dose to maximize tumor coverage and minimize facial nerve complications.

## Introduction

Paragangliomas are neuroendocrine neoplasms that arise most frequently in the head and neck. When these tumors arise from the jugular paraganglia, they are frequently referred to as glomus jugulare tumors (GJT) [1]. Due to their location, the spread of GJT often involves the lower cranial nerves (CNs) VII, IX, X, and XI, complicating treatment [2,3]. In cases of GJT involving lower CNs, resection carries a high risk of post-operative neurologic deficit, and thus stereotactic radiosurgery (SRS) is often the preferred treatment approach [4-6].

For the SRS treatment of GJT, single fraction dosage is often 13 to 15 Gy [7,8], with one group suggesting improved local control at or above 13 Gy [9]. However, previous reports indicate that the risk of permanent facial nerve paresis increases above 13 Gy [10]. Thus, there is a need to taper SRS dosages in areas of GJT involving the facial nerve to prevent a permanent neurologic deficit. To this end, “dose painting” can be useful to treat the vast majority of the tumor at full dose (at or above 15 Gy) while reducing the dose to the adjacent facial nerve to below 13 Gy [11]. This is often done on other body sites where the dose to the tumor is maximized and minimized on the surrounding organs at risk (OAR) [12].

Recently, the HyperArc (Varian Medical Systems, Palo Alto, CA) SRS treatment planning and delivery system has been introduced. HyperArc is a planning and delivery system for frameless linear accelerator (LINAC) volumetric modulated arc therapy (VMAT)-based SRS, incorporating several optimizations for treatment delivery speed while maintaining accuracy. Specifically, the system includes an automated multiple planar and non-coplanar arc dose planning interface, cone beam CT setup and optical surface monitoring of the patient’s position during delivery, and the use of flattening filter-free beams and automated couch movements during treatment. Multiple studies have reported its use for SRS of single and multiple brain metastases in 15-30 minutes [13-15]. For large and/or irregular base of skull targets, HyperArc can sculpt doses into irregular shapes while maintaining high delivery speed and accuracy.

The purpose of this report is to provide a rationale for the use of dose-painted SRS for GJT to provide maximum tumor control while preserving the facial nerve. To demonstrate this concept, we present a case series on the HyperArc system with a comparison of treatment plan quality and delivery time to the Gamma Knife (GK; Elekta Instrument AB, Stockholm, Sweden).

A preliminary version of this article was previously presented as a meeting poster at the 2022 NASBS Annual Meeting on February 17-19, 2023.

## Materials and methods

Patient selection

All nine patients included in this study were treated for GJT with HyperArc-VMAT-based SRS between 2019 and 2022 at the Sylvester Comprehensive Cancer Center at the University of Miami. The University of Miami Institutional Review Board approved the protocol (approval number: 20190678), and a retrospective review was conducted to gather demographic, tumor characteristics, radiation treatment, and follow-up data for all these patients. The requirement for patient consent was waived due to the retrospective study design.

Treatment planning

**Figure 1:**
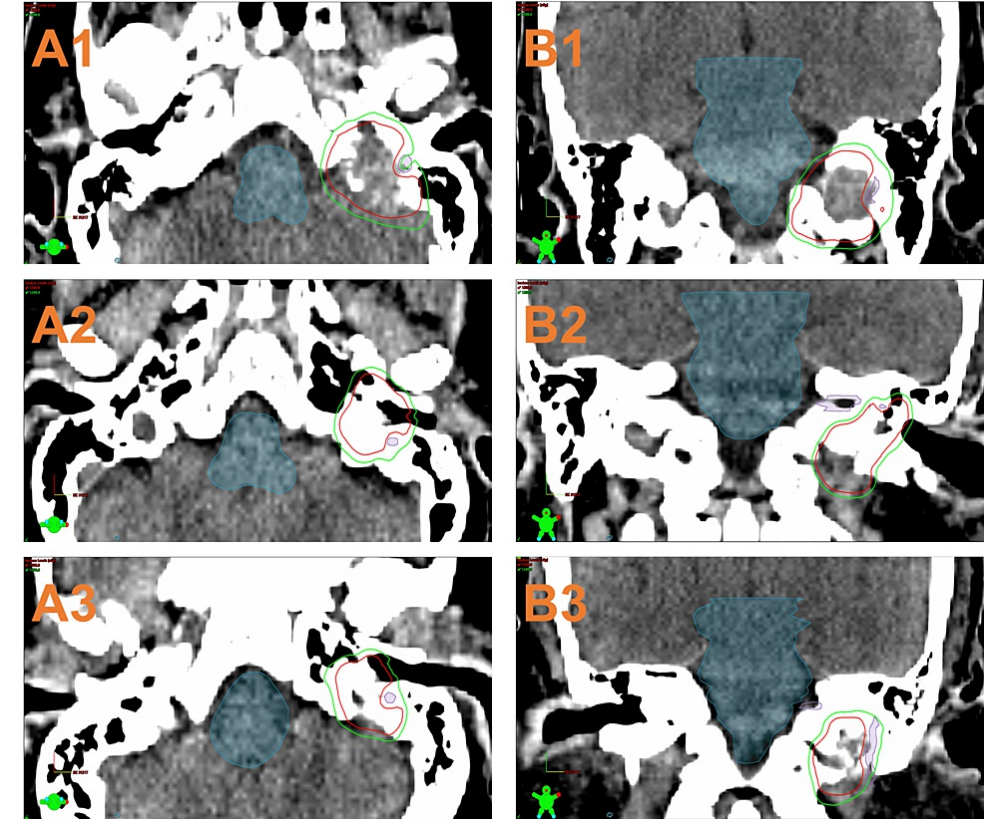
Treatment plans of three cases using dose-painted SRS HyperArc (A1, A2, and A3: axial view of Case 1, 2, and 3, respectively; B1, B2, and B3: coronal view of Case 1, 2, and 3, respectively) with isodose lines (red: 15 Gy; green: 12 Gy) and contours (blue: brainstem; purple: facial nerve). About 99% of the tumor is covered to 15 Gy in each case with a notch at the facial nerve where the dose is prescribed to 12 Gy

**Figure 2:**
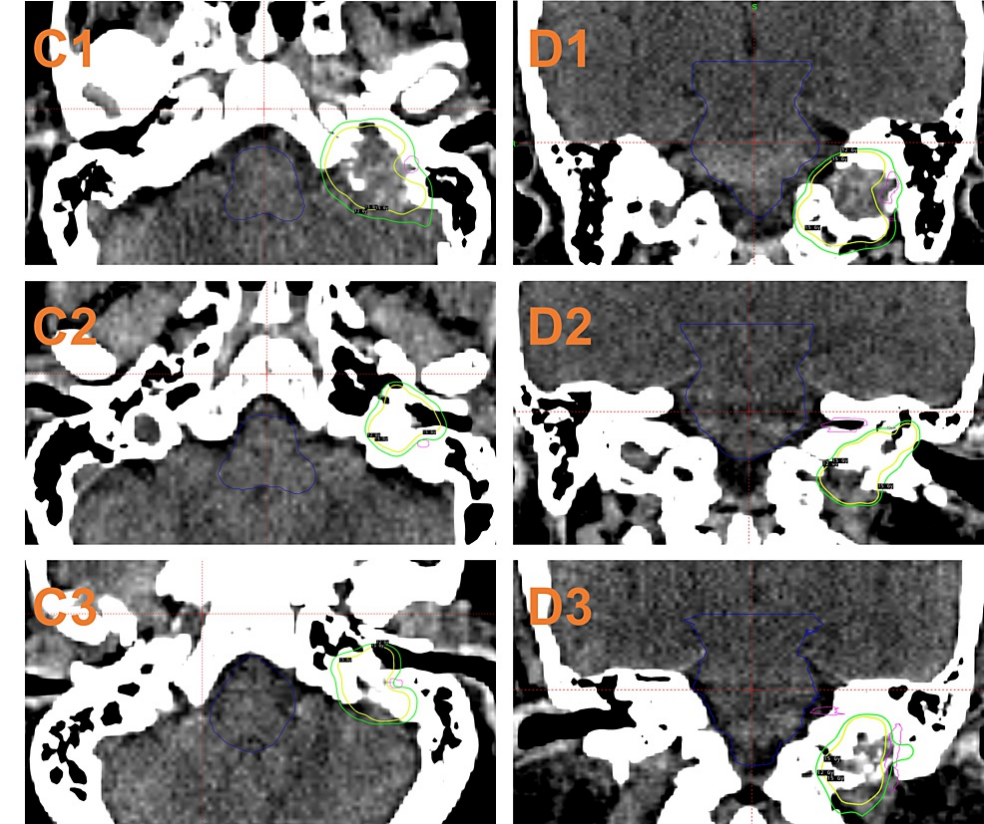
Treatment plans of three cases using GammaPlan (C1, C2, and C3: axial view of Case 1, 2, and 3, respectively; D1, D2, and D3: coronal view of Case 1, 2, and 3, respectively) with isodose lines (yellow: 15 Gy; green: 12 Gy) and contours (blue: brainstem; purple: facial nerve)

MRI and CT radiation planning sessions were performed for each patient in the days prior to the procedure. HyperArc SRS plans were generated to deliver the prescribed dose to the tumor plus a 1 mm planning target volume (PTV) in one fraction and an average prescription isodose of 67.5% (SD = 7.5%). When involved, the facial nerve was defined based on a 3D-T1-weighted MP-RAGE sequence acquired with the following parameters: TE (time to echo)/TR (time to repetition) = 3.02 ms/1650 ms, flip angle = 15º, acquisition matrix = 256$\times$192, voxel size = 1 mm$\times$1 mm$\times$1 mm, bandwidth = 179 Hz/Px, and acquisition time = eight minutes and 42 seconds. Any areas abutting CN VII were planned to be 12 Gy with a 1 mm margin around the nerve (Figure 1). Treatments were delivered with a 6MV energy level and a maximum dose rate of 1400 MU/min. All patients were treated with HyperArc, including an open-face thermoplastic mask system, a cone beam CT (CBCT) setup with adjustments by a 6-DOF couch, and facial optical surface monitoring. All treatment plans were performed by the same attending radiation oncologist to reduce intra-operative variation. Treatment plans for GK were generated retrospectively by using the automated planning system GammaPlan 11.3.2 (Elekta Instruments AB, Stockholm, Sweden) and additional manual planning to match the methods used for the respective HA cases based on the same planning MRI (Figure 2). All GK plans had the same dose rate of 2.348 Gy/min and an average prescription isodose of 51.6% (SD = 4.6%).

Dosimetric evaluation

Plan assessments of HyperArc and GK were performed by evaluating the conformity index (CI), homogeneity index (HI), and gradient index (GI) for each plan generated. Alternative definitions for CI and HI were included as the default index varies between planning systems.

CI is a measure of how closely the distribution of the prescribed dose matches the desired target. The Radiation Therapy Oncology Group (RTOG) defines CI as follows:

$$CI_{RTOG}= \frac{PIV}{TV }$$

where $PIV =$ prescription isodose volume and $TV =$ target volume.

An alternative definition proposed by Paddick [16] in 2000 is:

$$CI_{Paddick}= \frac{TV_{PIV}^{2}}{TV\times PIV}$$

where $TV_{PIV}=$ target volume covered by the prescription isodose.

HI is an objective tool to analyze the uniformity of dose distribution in the target volume. The RTOG describes it as:

$$HI_{RTOG}= \frac{D_{max}}{PI}$$

where $D_{max}=$ maximum isodose to the target and $PI=$ prescription isodose. The ideal value is 1, and it increases as the plan becomes less homogeneous.

An alternative definition for the calculation of HI was given by the International Commission on Radiation Units and Measurements Report 83 (ICRU83) as:

$$HI_{ICRU_{83}}= \frac{\left ( D_{2\%} -D_{98\%} \right )}{PI}$$

where $D_{2\%}$ and $D_{98\%}$ represent the isodose received by 2% and 98% of the tumor, respectively.

GI provides an indication of the dose falloff outside the target volume, where a lower GI value suggests a steeper dose gradient and potentially better sparing of surrounding healthy tissue. It has been described by Paddick and Lippitz [17] as:

$$GI = \frac{PIV_{50}}{PIV}$$

where $PIV_{50}=$ volume of the half prescription isodose.

Follow-up

Generally, patients followed up on the HyperArc SRS procedure at a scheduled visit with the attending radiation oncologist with a preceding MRI at 6, 12, 18, and 24 to 30 months after treatment. Documentation from these visits was reviewed to gather information regarding symptoms and posttreatment effects, including pulsatile tinnitus and CN function. Radiographic reports, in concomitance with their corresponding clinical notes, were reviewed to assess tumor response to treatment.

Statistical analysis

All data analysis was performed using R (R Core Team, 2023). Mean PTV coverage, treatment times, RTOG and Paddick CI, RTOG and ICRU83 HI, and GI were compared between HyperArc and simulated GK treatment plans using paired t-tests.

## Results

This series included nine patients: eight (89%) women and one (11%) man. All patients were diagnosed with a single GJT; six were left-sided and three were right-sided. The mean age at the time of treatment was 63.9 years (range, 41-85). All patients were diagnosed with GJT on the basis of clinical presentation and characteristic radiographic features. One patient in our series presented mild-to-moderate deficits of CNs VIII, IX, X, XI, and XII on the right side. No patients in our series demonstrated evidence of malignant lesions or catecholamine-secreting tumors. The mean pre-operative tumor volume was 12.4 cm^3^, and the median was 8.63 cm^3^ (Table 1).

**Table 1:** Summary of symptoms and tumor characteristics at presentation SD: standard deviation, N: number, CN: cranial nerve

Characteristics	Mean (SD) or N (%)
Total number of patients	9
Age at treatment (years)	63.9 (14.5)
Pre-Treatment Volume (cm^3^)	12.4 (14.7)
Sex	
Male	1 (11%)
Female	8 (89%)
Symptoms at presentation	
Pulsatile tinnitus	8 (89%)
Hoarseness	1 (11%)
Vertigo	5 (55.6%)
Hearing loss	9 (100%)
Otalgia	2 (22%)
Tongue deviation	1 (11%)
CN deficit	
None	8 (88.9%)
Single	0 (0%)
Multiple	1 (11.1%)
Tumor side	
Right	3 (33.3%)
Left	6 (66.7%)

**Figure 3:**
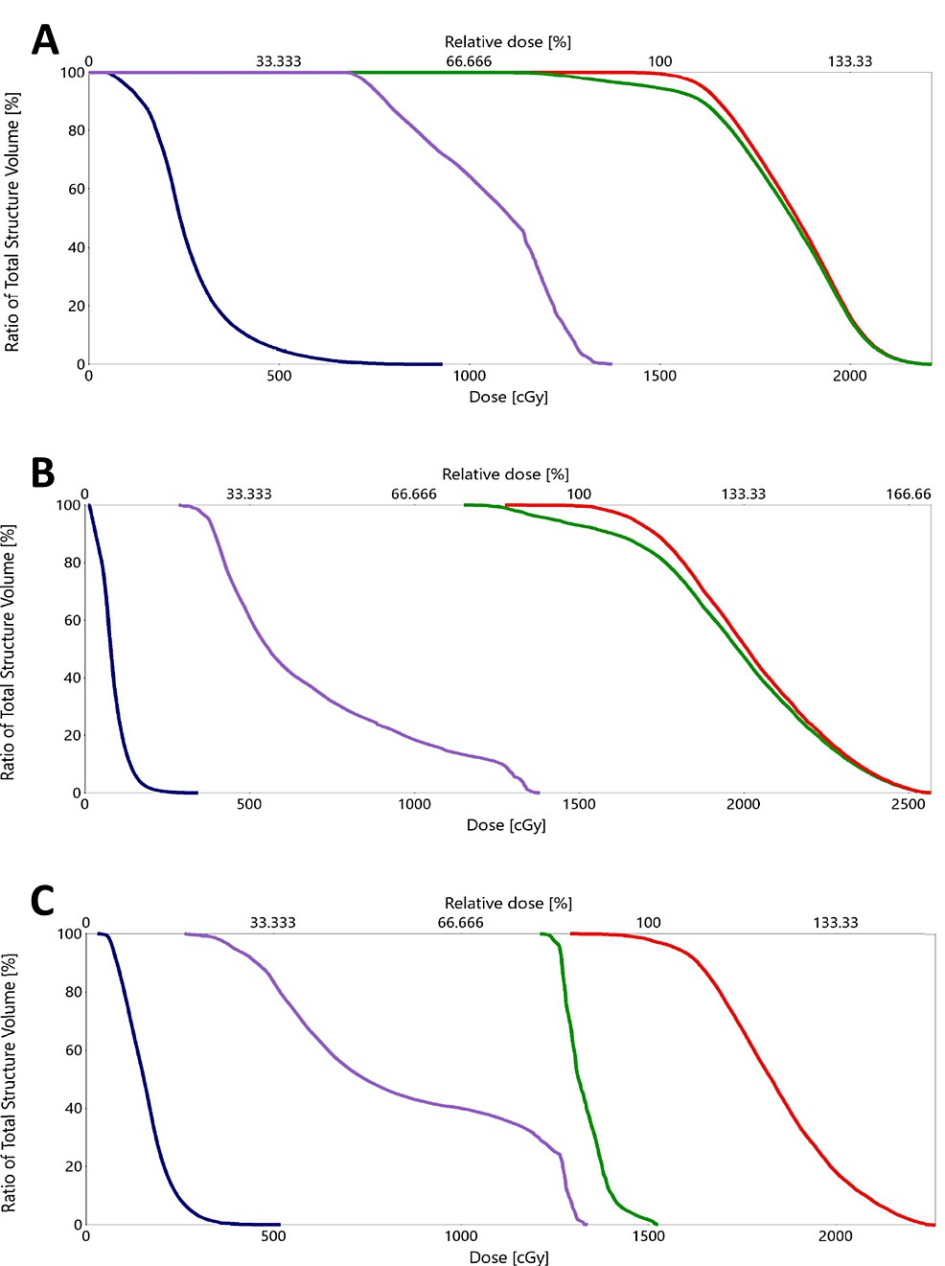
Dose volume histograms (A: Case 1, B: Case 2, C: Case 3). Lines represent: PTV15 (red), PTV12 (green), brainstem (blue), and facial nerve (purple). Less than 5% of the facial nerve receives 13 Gy or more (D0.03cc <13 Gy) with absolute maximum less than 14 Gy in all cases

The median marginal dose at the tumor periphery was 15 Gy (range 12-16 Gy). The maximum dose delivered ranged from 15.4 to 25.7 Gy (Table 2). In all cases (n=7) where the facial nerve was identified as an OAR, less than 0.03 cm^3^ received a dose greater than 13 Gy (Figure 3).

**Table 2:** Summary of SRS parameters delivered with HyperArc and duration of follow-up SD: standard deviation, SRS: stereotactic radiosurgery

Characteristics	Mean ± SD	Median	Range
SRS maximum dose (Gy)	20.7 ± 3.6	21.8	15.44-25.7
SRS margin dose (Gy)	13.8 ± 1.7	15	12-16
CN VII volume receiving >13 Gy (cc)	0.011 ± 0.009	0.015	0.0-0.021
Tumor Volume at time of SRS (cc)	12.4 ± 14.7	8.63	0.8-41.1
Radiographic follow-up after SRS (months)	18.8 ± 12.9	20	3-40

**Figure 4:**
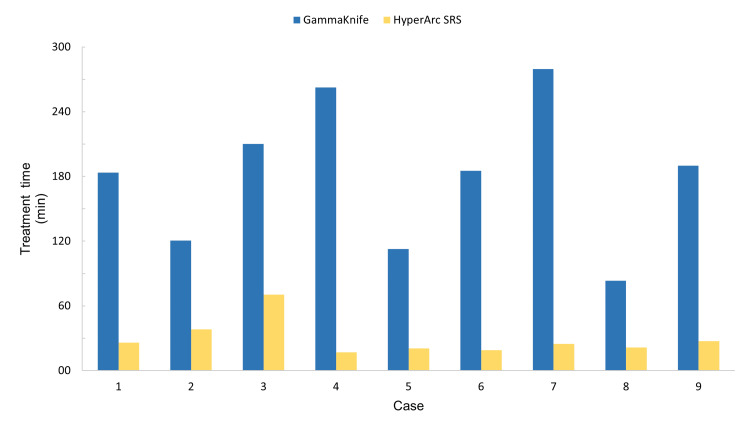
Comparison of HyperArc SRS and simulated GK plan total treatment times for each case included in this series GK vs. HyperArc treatment time SRS: stereotactic radiosurgery, GK: Gamma Knife

Treatment times and PTV coverage were compared between HyperArc and simulated GK modalities. The mean GK delivery time was 180 minutes, which exclusively included beam-on time. This was significantly longer than the average 29 minutes recorded for treatment using HyperArc (p=0.0001), which also included the time spent on quality assurance (QA) management and pre- and post-treatment CBCT performed to verify that all patients moved less than 1 mm or had less than 1° of rotation during the radiotherapy (Figure 4).

PTV coverage was intended to be 99.5%, with reductions below 99.5% due to sparing of facial nerves. GK plans achieved less mean PTV coverage than HyperArc despite unlimited use of mixed shots (20+ per plan) and blocking in attempts to spare facial nerve to ~13 Gy maximum dose (p=0.06).

**Figure 5:**
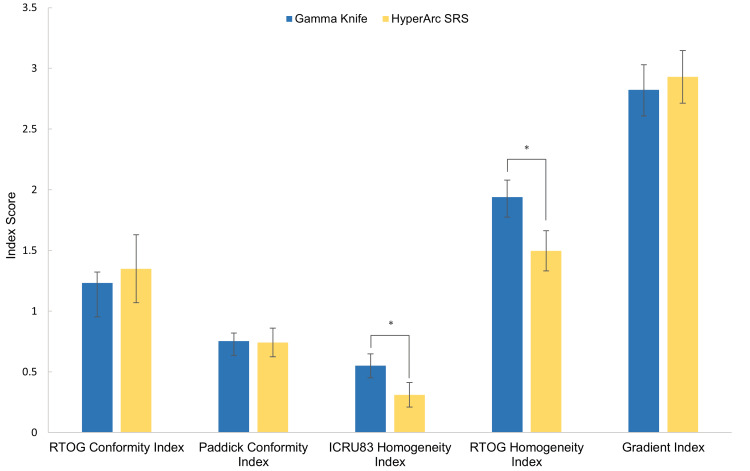
Dosimetric evaluation of delivered HyperArc SRS and simulated GK plans. Bars represent standard deviation and * indicates a significant difference (p<0.01) between the HyperArc and GK plans GK vs. HyperArc dosimetric evaluation SRS: stereotactic radiosurgery, GK: Gamma Knife

When comparing dosimetric parameters, there was no significant difference between the GI and CI values for the two different treatments. HyperArc plans always achieved a lower HI compared to the simulated GK plans (p=0.00004) (Figure 5, Table 3).

**Table 3:** Summary of time and dosimetric differences of the simulated GK plans compared to the delivered plan using HyperArc SD: standard deviation, GK: Gamma Knife, CI: conformity index, HI: homogeneity index, GI: gradient index * indicates a significant difference between GK and SRS HyperArc (p<0.01), ** indicates a strong trend (p=0.06)

Condition	Mean difference ± SD	Median	Range
Treatment time difference (min)*	151.42 ± 91.25	158	62-255
PTV coverage difference (%)**	-1.66 ± 2.3	-0.4	-5.4-1.6
RTOG CI	-0.12 ± 0.3	0.01	-0.62-0.15
Paddick CI	0.01 ± 0.1	0.02	-0.15-0.21
ICRU83 HI	0.25 ± 0.1	0.25	0.10-0.41
RTOG HI*	0.44 ± 0.2	0.49	0.18-0.66
GI	-0.11 ± 0.2	-0.23	-0.46-0.59

Follow-up

Clinical follow-up was available for the entire cohort of patients. A total of four (44.4%) patients experiencing hearing loss had improvement or resolution of symptoms. Fifty percent of the eight patients who had pulsatile tinnitus reported improvement or resolution, and 60% of the five patients with vertigo reported improvement. Only one patient reported temporary worsening after treatment with left V1 and V2 facial numbness, which was suspected to be due to radiation hypersensitivity, and areas of enhancement outside of the high-dose radiation field (12 Gy), suspected to be posttreatment effects, were observed at the two-month follow-up MRI. Both facial numbness symptoms and radiographical enhancement spontaneously resolved without further intervention. Coincidentally, this was the only subject who received a margin dose of 16 Gy for the tumor not affecting the facial nerve. All other pre-SRS-reported symptoms remained stable on follow-up for all patients.

Follow-up imaging with a contrast-enhanced MRI was available for all nine subjects. The mean radiographical follow-up was 18.8 months, and the median was 20 months. A tumor size decrease was observed in one (11.1%) patient, and for all other subjects, the size of the treated tumor remained unchanged.

## Discussion

The ideal therapeutic approach to GJT remains controversial due to the high morbidity associated with surgery [18]. A meta-analysis conducted by Ivan et al. suggested SRS conferred improved tumor control with lower rates of neurologic morbidity compared to gross total resection [4]. At our institution, we recommend radiation as a first-line treatment for minimally symptomatic GJT involving the jugular bulb. Surgery for GJT is favored when there is a pre-existing lower CN deficit, functional tumors (i.e., adrenergic hypersecretion), significant pain and airway compromise from a large tumor burden, imminent intracranial complications, and suspicion of malignancy, which is raised by the following features: the presence of nodal metastasis, accelerated tumor growth, and mutations in oncogenes such as the subunit B of the succinyl dehydrogenase gene and of the Von Hippel-Lindau gene.

The correlation of SRS treatment dose with the tumor recurrence rate of GJT is controversial. Chen et al. demonstrated that GK doses of 15 Gy or more were associated with a higher rate of treatment success [9]. However, others have found dosages less than 15 Gy to be sufficient for tumor control [7,19]. Given the limited evidence, it seems prudent to maintain doses of 15 Gy or more to obtain the best GJT control when it is safe to do so [20-22].

While facial nerve toxicity has been reported after SRS of GJT, it is better described in the SRS treatment of vestibular schwannomas since they are more common and always about the facial nerve anatomically (Table 4) [23]. The risk of facial paresis in GK treatment of vestibular schwannomas was greater in patients treated at doses of 15 Gy or higher, while doses less than 13 Gy were associated with preserved function [20-22,24]. The true tolerance of the facial nerve cannot be known since the exact relationship of the nerve to GK plan hot spots cannot be identified. For safety, when treating SRS at the skull base, we limit the facial nerve to 15 Gy maximum point dose and attempt for small volumes at 13 Gy or more, as we have presented in this article.

**Table 4:** Facial nerve toxicity outcomes at different dosages described in the literature for SRS treatment of GJT and vestibular schwannoma GJ: glomus jugulare, VS-FN: vestibular schwannoma abutting the facial nerve, N: total sample size, SRS: stereotactic radiosurgery, GJT: glomus jugulare tumor

Pathology	Study	Year	N	Dosage tested	Size measurement	Tumor size	Mean follow-up (months)	Outcome parameters	Results
GJ	Sharma et al. [19]	2018	42	>15 Gy vs <15 Gy	Median volume, maximal diameter	5 cm^3^, 3 cm	62.3	Local control	No difference in tumor control
GJ	Winford et al. [7]	2017	38	13.2 Gy vs 13.7 Gy	Mean maximal diameter	5.8 cm	39.1	Local control	Higher risk of progression with increased dosage
GJ	Chen et al. [9]	2010	15	14.6 Gy vs 13.2 Gy	Mean volume	7.3 cm^3^	43.2	Local control	Higher risk of treatment failure with a lower dose
VS-FN	Lerner et al. [24]	2020	133	15.49 Gy vs 12.42 Gy	Mean maximal diameter	1.64 cm	17.5	Complications	Higher risk of facial paresis with a higher dose
VS-FN	Sheehan et al. [22]	2015	42	≤12.5 Gy vs >12.5 Gy	Mean volume	≤1 cm^3^	28	Complications	Higher risk of permanent neurologic deficit with a higher dose
VS-FN	Hasegawa et al. [20]	2013	347	>13 Gy vs <13 Gy	Median volume	2.8 cm^3^	150	Complications	Higher risk of facial paresis with a higher dose
VS-FN	Yang et al. [21]	2009	2204	>13 Gy vs <13 Gy	N/A	N/A	54.1	Complications	Higher risk of facial paresis with a higher dose

Several approaches are available for SRS treatment of GJT, including VMAT, a relatively novel technique allowing for variation of gantry rotation speed, treatment aperture shape, and dose rate for improved PTV coverage with sparing of normal tissues [25]. However, non-coplanar LINAC-based SRS delivery may be complex and time-consuming. HyperArc simplifies SRS delivery by achieving full automation of non-coplanar SRS treatment planning and delivery that allows for irradiation of multiple targets without the need for patient repositioning or the generation of separate treatment plans per target. Unlike GK, the HyperArc system is able to easily transition between radiating with different doses and allows for significantly less beam-on time [26], with similar or better results than GK in the treatment of multiple brain tumors [13,15,27-32]. Together, this allows for shorter treatment times. Indeed, for our small sample of nine cases, HyperArc significantly reduced mean treatment times compared to GK by 151 minutes (2.5 hours) with dosimetrically equivalent plans, with the exception of HI. It is unclear whether this difference in HI translates into differences in clinical outcomes. We believe this difference to be solely due to the distinct treatment delivery mode of each platform.

Strengths and limitations

This study is susceptible to all biases inherent in retrospective, non-randomized designs. Future investigations comparing GK and HyperArc planning and delivery of SRS should ideally employ prospective, randomized assignment to either treatment modality. Though our sample size was modest, we still identified a significant difference in treatment time and HI between the two SRS modalities. PTV coverage trended toward greater coverage with HyperArc planning, though this was not statistically significant, which may be due to a lack of statistical power. We will continue to explore the role of the HyperArc system in the treatment of GJT as our sample expands and the data matures.

## Conclusions

We present a technique for the treatment of GJT using the recently introduced HyperArc radiosurgery system. The system performs radiosurgery of skull base tumors like GJT in ~29 minutes. It also allows for the delivery of multiple radiotherapy dose levels, and we demonstrate our rationale and technique for escalating doses to GJT with a reduction of dose to preserve the function of the involved facial nerve. Compared to GK, treatment delivery is several hours faster with the ability to deliver two dose levels expeditiously. Future investigations comparing GK and HyperArc planning and delivery of SRS should ideally employ prospective, randomized assignment to either treatment modality. Long-term exploration of the HyperArc system for the treatment of GJT is planned.
